# Signal Quality Assessment of a Novel ECG Electrode for Motion Artifact Reduction

**DOI:** 10.3390/s21165548

**Published:** 2021-08-18

**Authors:** Hesam Halvaei, Leif Sörnmo, Martin Stridh

**Affiliations:** Department of Biomedical Engineering, Lund University, SE-22100 Lund, Sweden; martin.stridh@bme.lth.se

**Keywords:** wet ECG electrode, motion artifacts, signal quality assessment

## Abstract

Background: The presence of noise is problematic in the analysis and interpretation of the ECG, especially in ambulatory monitoring. Restricting the analysis to high-quality signal segments only comes with the risk of excluding significant arrhythmia episodes. Therefore, the development of novel electrode technology, robust to noise, continues to be warranted. Methods: The signal quality of a novel wet ECG electrode (Piotrode) is assessed and compared to a commercially available, commonly used electrode (Ambu). The assessment involves indices of QRS detection and atrial fibrillation detection performance, as well as signal quality indices (ensemble standard deviation and time–frequency repeatability), computed from ECGs recorded simultaneously from 20 healthy subjects performing everyday activities. Results: The QRS detection performance using the Piotrode was considerably better than when using the Ambu, especially for running but also for lighter activities. The two signal quality indices demonstrated similar trends: the gap in quality became increasingly larger as the subjects became increasingly more active. Conclusions: The novel wet ECG electrode produces signals with less motion artifacts, thereby offering the potential to reduce the review burden, and accordingly the cost, associated with ambulatory monitoring.

## 1. Introduction

The presence of noise continues to plague the analysis and interpretation of the ECG, particularly salient in recordings obtained under ambulatory conditions. The most straightforward approach to dealing with noise is to employ a signal quality index (SQI), which, in its simplest form, serves as a binary flag indicating whether or not a signal segment should be excluded from further analysis. In its more advanced form, the SQI provides information on the level of usefulness of the ECG, e.g., whether suitable for diagnostic purposes, rhythm analysis, heart rate computation, or unsuitable for further analysis. Using signal quality assessment, the analysis obviously becomes more reliable, but at the expense of excluding signal segments. Especially in noisy ambulatory recordings, exclusion can become quite substantial, leading to that intermittent arrhythmias can go undetected. Signal quality assessment has in recent years become an art in itself as evidenced by the great number of studies [1,2,3,4,5,6,7,8,9,10,11,12].

Another approach is to denoise the ECG using nonlinear signal processing techniques, e.g., nonlinear Bayesian filtering [13], adaptive filtering [14,15,16], empirical mode decomposition [17], multiresolution thresholding using the discrete wavelet transform [18,19] or the empirical wavelet transform [20]. The design of denoising techniques is challenged by the fact that the spectral content of motion artifacts, as well as that of muscle noise, overlaps considerably with that of the QRS complex [21], thus implying that linear filtering techniques cannot be used. While denoising may be performed in ambulatory recordings aimed for rhythm analysis, there is a risk that denoising distorts the diagnostic information of ECG recordings aimed for analysis of beat morphology [13]. So far, performance evaluation of denoising techniques has been expressed in engineering terms, e.g., signal-to-noise ratio [14,15,16,20], rather than in clinical terms reflecting changes in, e.g., wave amplitude and duration.

The direct way to improve signal quality is by innovation in ECG electrode technology, especially with regard to motion artifacts as they are caused by deformation of the skin close to and under the electrode and external forces on the electrode from adjacent objects, e.g., electrode lead and clothing [22]. Motion artifacts are manifested as large-amplitude waveforms often mistaken for QRS complexes, leading to false detection of arrhythmias, notably atrial fibrillation (AF). In turn, this leads to a time-consuming manual review load. With regard to muscle noise, being the other major noise source, its bioelectrical origin renders innovation in electrode technology much more difficult.

Recent research on ECG electrodes has mostly been focused on the development of dry ECG electrodes [23,24,25,26,27,28]. In contrast to conventional wet/gel electrodes, dry electrodes have the important advantage of not requiring electrolytic conductive gel and skin preparation to reduce the skin–electrode contact impedance. Still, after many years of research on dry electrodes, wet electrodes continue to remain the standard in clinical applications thanks to better signal quality, whereas dry electrodes are more prone to motion artifacts due to less strong skin fixation and higher skin–electrode impedance [23].

Similar results have been reported in studies comparing the quality of EEG signals acquired by dry and wet electrodes [29,30]: Dry electrodes were found to exhibit a marked increase in the level of broadband noise [29], and a statistically significantly lower percentage of artifact-free segments [30].

Both visual [23,26,31] and quantitative [25,27,31,32] approaches have been considered for assessment of electrode signal quality. The assessment has been based on just one single subject [23,26], but also on larger datasets including 25–30 subjects [31,32]. When a quantitative approach is pursued, indices like the root mean square value, the peak-to-peak amplitude of the signal averaged QRS complex, and the Pearson’s correlation coefficient have been employed to characterize signal quality.

The aim of the present study is to assess a novel wet ECG electrode with regard to its ability to reduce the presence of motion artifacts. The assessment is based on ECG signals acquired during different types of physical activity, primarily reflecting the short-term properties of the electrode. Signal quality is quantified in terms of sensitivity and positive predictive value of QRS detection, false positive rate of AF detection, and two different indices originally proposed for exclusion of noisy ECG segments. The performance of the electrode is compared to that of a commercially available electrode.

## 2. Materials

### 2.1. Electrode Technology

The wet Piotrode^®^ electrode (Piotrode Medical AB, Stockholm, Sweden) is currently a prototype designed to reduce electrode motion artifacts of electrostatic and mechanical origins. Electrostatic disturbances are reduced by the combined effect of a two-layer shielding structure, comprised of an electrostatic dissipative layer and an electrical conductive layer, and a skin contacting element, comprised of an ion conductive substrate connected to the skin. The shielding structure and the skin contacting element are electrically connected. The structure reduces electrostatically induced voltages on the electrode caused by clothing and other external objects. The induced voltages may, if not properly reduced, cause noise in the ECG signal.

To reduce disturbances of mechanical origin, a stabilizing structure is introduced to protect the conductive compartment, i.e., the wet gel and the Ag/AgCl sensor part, from mechanical disturbances. The stabilizing structure is comprised of a bistable lid covering/circumventing the wet gel compartment and four evenly distributed air evacuation channels connecting the wet gel compartment to the outer surface of the electrode, forming a fluid connection between the wet gel compartment and the ambient air. The bistable lid has an upper stable position and a collapsed/lower position. Once the electrode is attached to the body, a gentle pressure with the index finger on the bistable lid changes the position of the lid from the upper position to the collapsed position thereby evacuating residual trapped air which if present may move around and influence the skin/electrolyte interface and thereby cause electric disturbances. The dimensions and mechanical properties of the air evacuation channels are designed with respect to the viscoelastic properties of the wet gel in such a way that no gel flows through the channels. Thanks to the stabilizing structure, the volume of the conductive compartment maintains its structural integrity when subjected to external forces, thereby reducing the amount of electrical disturbances generated in the skin/electrolyte interface during deformation.

For comparison, the wet electrode Ambu^®^ BlueSensor L (Ambu, Copenhagen, Denmark) is used with an adhesive on the skin-contacting surface of the electrode. It is noted that this electrode was used as a reference in a recent, comparative study [32].

**Figure 1:**
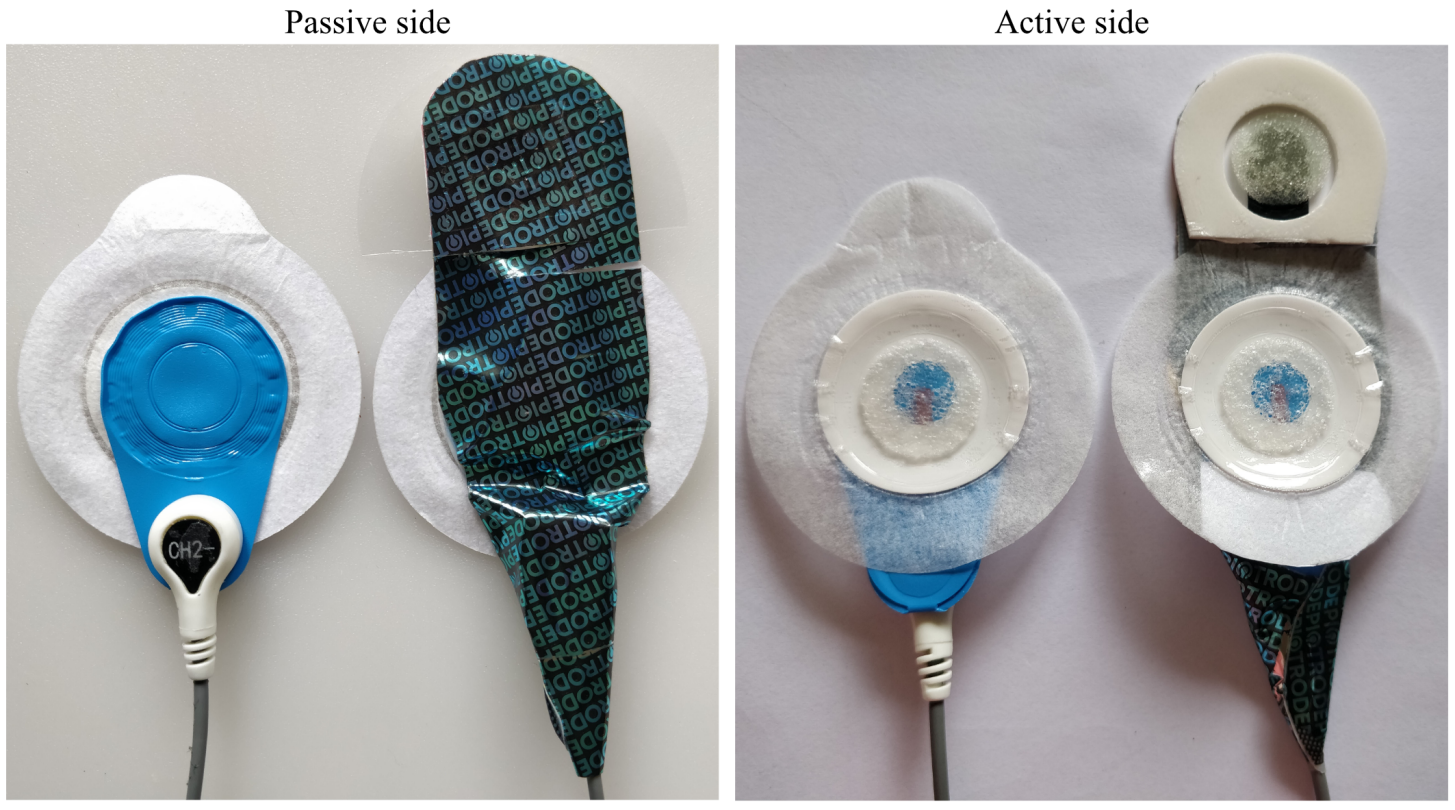
The Ambu BlueSensor L (**left**) and the Piotrode electrode (**right**).

Both the Piotrode and the Ambu BlueSensor L have an oval shape with a width of 55 mm, whereas their respective lengths are 82 mm and 62 mm, see Figure 1. For both electrodes, the connector for attaching the lead is positioned at an offset distance of 25 mm from the center of the electrode.

### 2.2. Electrode Placement and Data Acquisition

**Figure 2:**
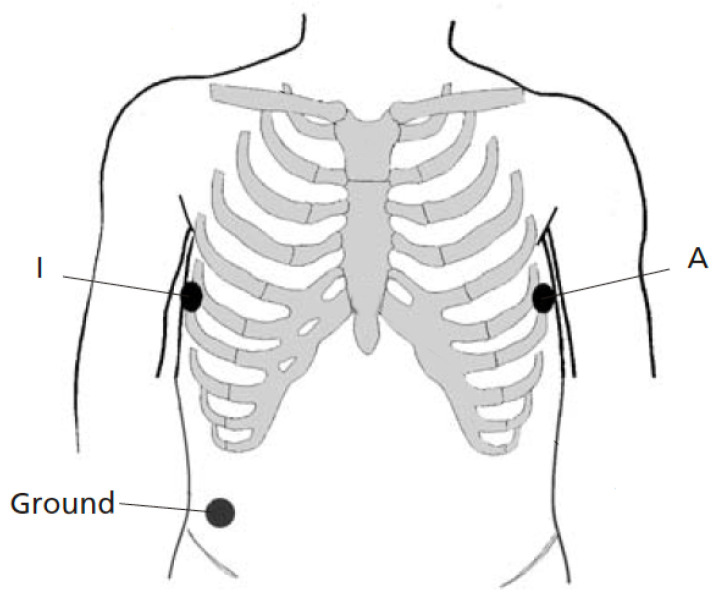
Lead placement for recording two simultaneous single-lead ECGs (lead AI). The two electrodes for comparison are positioned as closely to each other as possible at the positions A and I. The same ground electrode is used for both electrodes.

Figure 2 shows the electrode placement used for recording two simultaneous single-lead ECGs. The two electrodes were positioned as closely to each other as possible at A and I, that is, two positions which are part of the EASI lead system [33], see also [32]. The positions were placed on the left and right midaxillary lines at the same height. Thus, lead AI views the electrical activity of the heart in a left-to-right direction. To avoid favoring the Piotrode, lead AI was chosen for signal quality assessment as it tends to be less affected by motion artifacts than leads positioned higher up on the chest [34]. The same ground electrode was used by both electrodes.

In an effort to record the ECG under similar conditions, the relative positions of the electrodes at A and I alternated from subject to subject. No preconditioning/abrasion was made before attaching the electrodes.

The ECG was acquired using Amedtec ECG Pro Cardiopart 12 Blue Holter Recorder device (AMEDTEC Medizintechnik Aue GmbH, Aue, Germany), digitized at a sampling rate of 500 Hz. Two leads were recorded simultaneously; thus a total of five electrodes were attached to the body surface.

### 2.3. ECG Dataset and Annotation

20 healthy subjects (4 females and 16 males aged between 24 and 77 years) were enrolled in the study. The ECG was recorded continuously during one single session involving six different activities, performed in an order defined by the following protocol:

1. Sitting at rest
2. Sitting and crossing arms
3. Walking on floor
4. Walking in stairs
5. Running
6. Undressing and dressing

To ensure adherence to the protocol, a coach informed each subject about the next activity and related timing. Each activity was performed at a moderate, steady pace during 2 min, except the first two activities which were performed during 1 min. To avoid that transitioning from one activity to another influenced the ECG signal, a 15-s pause was inserted between successive activities; the pause was excluded when assessing signal quality. No recommendation on what to wear was disclosed in advance, but the subjects wore their everyday clothing.

Since some of the SQIs employed in the present study require information on heartbeat occurrence time, the dataset was annotated using a semi-automated approach in which QRS detection was performed in the lead exhibiting the better signal quality (visually determined). The detected events were then manually reviewed to ensure that no false detections were included in the series of annotated occurrence times.

## 3. Methods

Signal quality is assessed in terms of detection performance, i.e., detection of QRS complexes and false detection of AF episodes, and indices quantifying different characteristics of noise and artifacts, described in Section 3.1 and Section 3.2, respectively. Since QRS detection is required when computing the SQIs below, detection performance is considered first in the following.

### 3.1. Detection Performance

QRS detection is accomplished using a well-performing and thoroughly evaluated wavelet-based detector [35]. Its performance is characterized in terms of sensitivity (Se) and positive predictive value (PPV), defined as follows:

(1) $$\begin{array}{c} \mathrm{Se}=\frac{N_{\mathrm{TP}}}{N_{\mathrm{TP}}+N_{\mathrm{FN}}}, \end{array}$$

(2) $$\begin{array}{c} \mathrm{PPV}=\frac{N_{\mathrm{TP}}}{N_{\mathrm{TP}}+N_{\mathrm{FP}}}, \end{array}$$

where $N_{\mathrm{TP}}$ is the number of correctly detected QRS complexes, $N_{\mathrm{FN}}$ is the number of missed QRS complexes, and $N_{\mathrm{FP}}$ is the number of falsely detected QRS complexes; all performance indices are computed subject-wise. Any occurrence time produced by the QRS detector deviating less than 100 ms from the annotated occurrence time is deemed to be correct. The overall Se and PPV, presented below, are computed as averages across subjects.

Based on the output of the QRS detector, rhythm-based AF detection is performed in which the irregularity of the resulting RR interval series is analyzed [36]. A well-performing, low-complexity AF detector is employed incorporating blocks for ectopic beat filtering, bigeminy suppression, characterization of RR interval irregularity, and signal fusion [37]; see also [36] for a comparison of AF detector performance. Since the dataset contains only healthy subjects, the false positive rate (FPR) can only be assessed, whereas other statistical performance indices such as the above-mentioned Se and PPV cannot. The FPR is defined by

(3) $$\mathrm{FPR}=\frac{N_{\mathrm{FP}}}{N_{\mathrm{FP}}+N_{\mathrm{TN}}},$$

where $N_{\mathrm{FP}}$ is the number of falsely detected beats in AF and $N_{\mathrm{TN}}$ is the number of correctly identified beats in normal sinus rhythm.

### 3.2. Signal Quality Indices

Before computing the different SQIs, the ECG signals are preprocessed for the purpose of removing baseline wander, using a third-order Butterworth highpass filter (cutoff frequency of 0.5 Hz) in combination with forward–backward filtering [21]. Moreover, ectopic beats with aberrant morphology are excluded.

By forming an ensemble of the events, aligned with reference to their occurrence times as produced by the QRS detector, the ensemble standard deviation is computed, defined by

(4) $$\sigma_{e}\left(n\right)=\sqrt{\frac{1}{M}\sum_{i=1}^{M}{\left(x_{i}\left(n\right)-{\bar{s}}_{a}\left(n\right)\right)}^{2}},\;n=0,\cdots ,N-1,$$

where *M* is the number of events in the ensemble (determined by the QRS detector), $x_{i}\left(n\right)$ is the *n*:th sample of the *i*:th event, *N* is the number of samples of each event, and ${\bar{s}}_{a}\left(n\right)$ is the ensemble average. The onset of the interval [0,N−1] is positioned 150 ms before the occurrence time, and *N* is set to 400 ms. Since the ensemble standard deviation not only reflects noise and artifacts but, to a certain degree, also respiration [38,39], min-max normalization of $x_{i}\left(n\right)$ is performed before $\sigma_{e}\left(n\right)$ is computed. To facilitate interpretation, the SQI is defined as 1 min the time average of the ensemble standard deviation $\sigma_{e}\left(n\right)$,

(5) $${\bar{\sigma }}_{e}=1-\frac{1}{N}\sum_{n=0}^{N-1}\sigma_{e}\left(n\right).$$

Thus, as ${\bar{\sigma }}_{e}$ becomes increasingly closer to 1, the signal quality becomes increasingly better.

Another approach to assessing signal quality is to quantify the repeatability of successive events in the time–frequency domain. Accordingly, this is a more sophisticated approach than the ensemble standard deviation in the sense that it accounts for morphology [7]. A time–frequency representation of $x_{i}\left(n\right)$ is obtained using the smoothed pseudo Wigner–Ville transform [40],

$$X_{i}\left(n,k\right),\;n=0,\cdots ,N-1;k=0,\cdots ,K-1,$$

where *K* is the number of frequency samples. The quality of $X_{i}\left(n,k\right)$ is determined by how well it correlates with a time–frequency template ${\bar{X}}_{i}\left(n,k\right)$, taken as the average of the three preceding events, i.e., $X_{i-1}\left(n,k\right),X_{i-2}\left(n,k\right)$, and $X_{i-3}\left(n,k\right)$. The weighted Pearson correlation coefficient is employed to quantify repeatability, defined by

(6) $$\begin{array}{c} \rho_{i,j}=\frac{\sum_{n=0}^{N-1}\sum_{k=0}^{K-1}w\left(n,k\right)X_{i-j}\left(n,k\right){\bar{X}}_{i}\left(n,k\right)}{\sqrt{\sum_{n=0}^{N-1}\sum_{k=0}^{K-1}w\left(n,k\right)X_{i-j}^{2}\left(n,k\right)}\sqrt{\sum_{n=0}^{N-1}\sum_{k=0}^{K-1}w\left(n,k\right){\bar{X}}_{i}^{2}\left(n,k\right)}}, \end{array}$$

where both $X_{i-j}\left(n,k\right)$ and ${\bar{X}}_{i}\left(n,k\right)$ have been mean-centered. The weight function w(n,k) is defined by three Gaussian functions center-positioned in intervals where the P wave, the QRS complex, and the T wave are expected to occur in normal sinus rhythm, see [7] for details. The overall signal quality of an ECG recording is obtained by first averaging $\rho_{i,j}$ over the four most recent events, yielding the SQI of the *i*:th event, followed by averaging over all *M* events,

(7) $$\bar{\rho }=\frac{1}{M}\sum_{i=1}^{M}\left(\frac{1}{4}\sum_{j=0}^{3}\rho_{i,j}\right).$$

As $\bar{\rho }$ becomes increasingly closer to 1, the overall signal quality of the recording becomes increasingly better.

## 4. Results

### 4.1. Detection Performance

**Figure 3:**
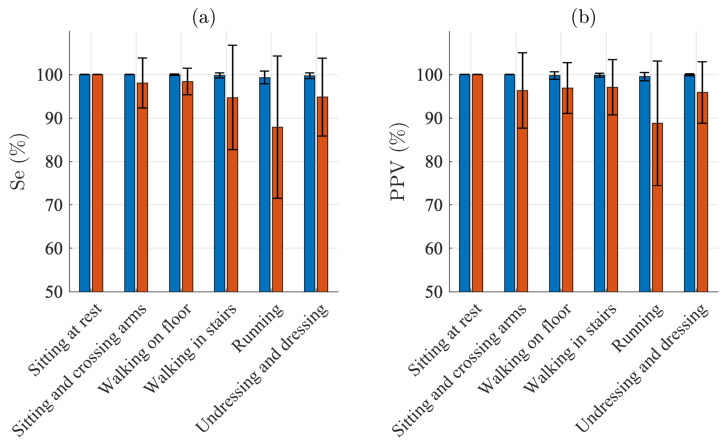
QRS detection performance: mean and standard deviation of (**a**) sensitivity and (**b**) positive predictive value of the Piotrode (blue) and the Ambu (red) electrodes.

Figure 3 presents the QRS detection performance for each of the different activities. For sitting, Se and PPV are 100% for both electrodes. For the other five activities, Se ranges from 99.3% to 100.0% and PPV from 99.5% to 100.0% for the Piotrode, whereas, for the Ambu, Se ranges from 87.9% to 98.4% and PPV from 88.8% and 97.1%. As expected, using either of the electrodes, the lowest performance figures are obtained for running. The subject-to-subject variation in performance is considerably larger for the Ambu than for the Piotrode: For running, the standard deviation of Se is 1.4% and 16.4% for the Piotrode and the Ambu, respectively, whereas the standard deviation of PPV is 0.9% and 14.3%, respectively.

**Figure 4:**
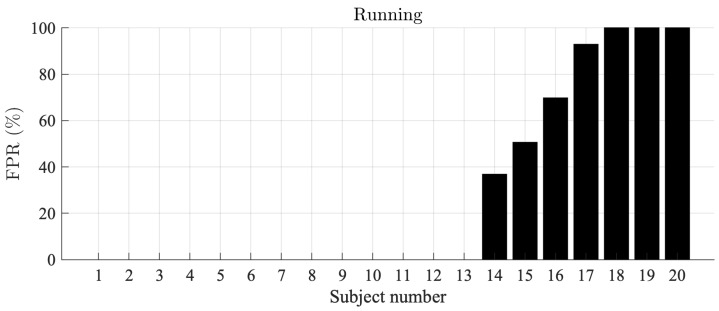
AF detection false positive rates during running, sorted in ascending order, for the Ambu electrode. The false positive rate for the Piotrode electrode was 0.0% in all subjects.

For the Piotrode, no false AF detections are observed during running, i.e., the most physically demanding activity. On the other hand, for the Ambu, AF is falsely detected in 7 out of 20 subjects, with an FPR ranging from 36% to 100%, see Figure 4.

**Figure 5:**
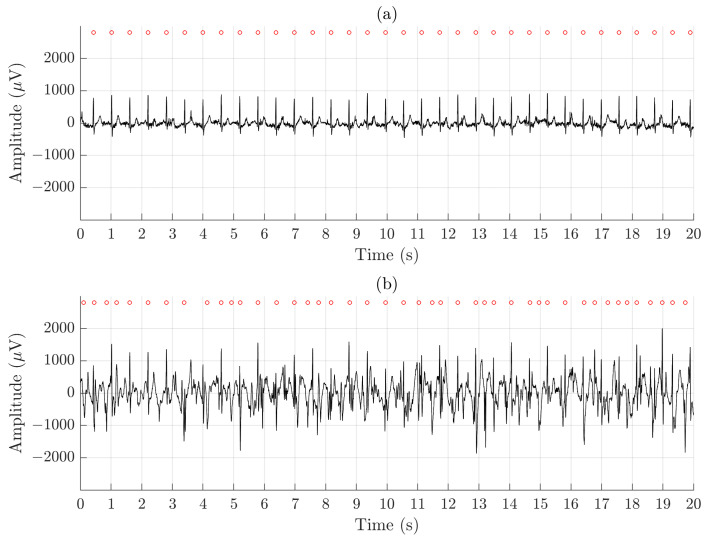
An example of signal recorded simultaneously during running using (**a**) the Piotrode and (**b**) the Ambu electrodes.

Figure 5 illustrates the difference in signal quality between the Piotrode and the Ambu during running. For this particular subject, using the Ambu, Se and PPV of the QRS detector are 86.2% and 73.5%, respectively, and FPR of the AF detector is 50.6%; the corresponding figures for the Piotrode are 100.0%, 100.0% and 0%. The difference in morphology observed between the two ECG signals is due to the edge-to-edge electrode location.

### 4.2. Signal Quality Indices

**Figure 6:**
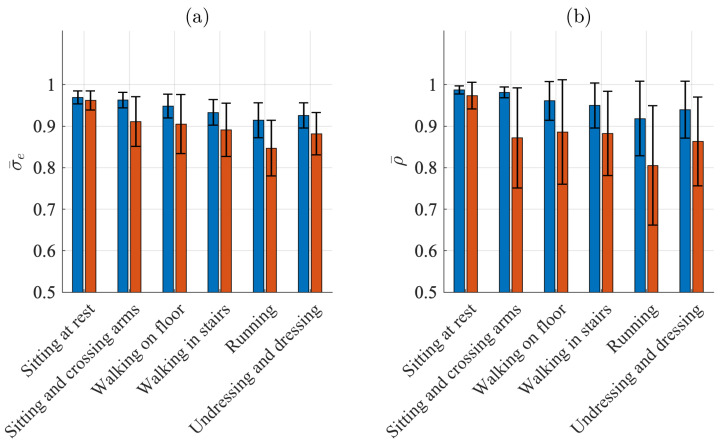
Mean and standard deviation of (**a**) ${\bar{\sigma }}_{e}$ and (**b**) $\bar{\rho }$ for the Piotrode (blue) and the Ambu (red) electrodes.

Figure 6a,b present the results when signal quality is characterized by ${\bar{\sigma }}_{e}$ and $\bar{\rho }$, respectively. Similar to the detection performance indices, the SQIs indicate the best quality when sitting (${\bar{\sigma }}_{e}=0.97$ and $\bar{\rho }=0.99$ for the Piotrode and ${\bar{\sigma }}_{e}=0.96$ and $\bar{\rho }=0.97$ for the Ambu) and the worst quality when running (${\bar{\sigma }}_{e}=0.91$ and $\bar{\rho }=0.92$ for the Piotrode and ${\bar{\sigma }}_{e}=0.85$ and $\bar{\rho }=0.80$ for the Ambu). For all activities except sitting, the Piotrode offers better signal quality than does the Ambu.

The subject-to-subject variation in signal quality is considerably larger for the Ambu than for the Piotrode for most activities, especially when signal quality is characterized by $\bar{\rho }$ during sitting and crossing arms and walking.

## 5. Discussion

In the last decade, substantial efforts have been directed toward the development of signal processing techniques for removal of motion artifacts, by some considered as the most problematic noise source in ambulatory ECG recordings [41]. Such techniques may improve the reliability of arrhythmia analysis and allow the usage of inexpensive standard electrodes, but unfortunately at the expense of signal distorsion which may be unacceptable in diagnostic applications. In the present study, a novel ECG electrode specifically designed to reduce motion artifacts of electrostatic and mechanical origins has been assessed.

**Figure 7:**
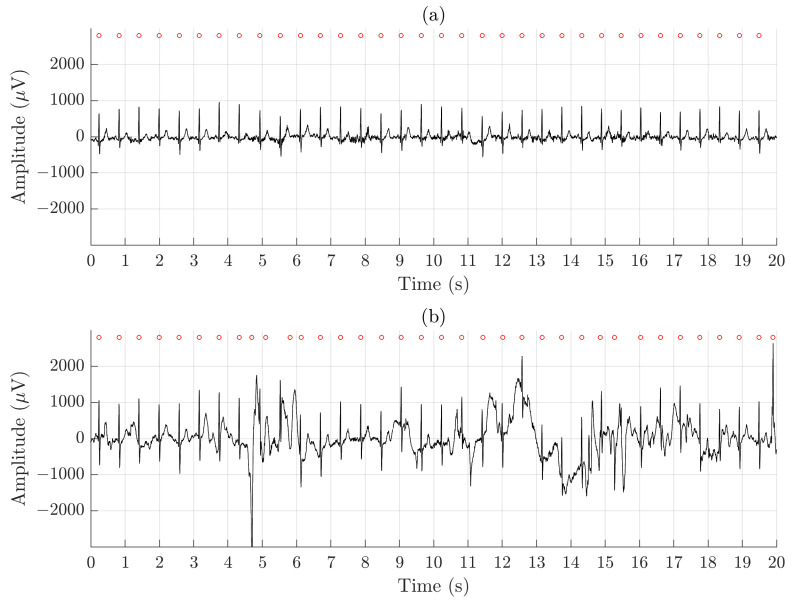
An example of signal recorded simultaneously during undressing and dressing using (**a**) the Piotrode and (**b**) the Ambu electrodes.

Using QRS detection performance as an indicator of whether arrhythmia analysis is feasible to perform, the results show clearly that the ECGs recorded by the Piotrode is more feasible for such analysis, even during running. Using the Ambu, falsely detected QRS complexes due to motion artifacts were observed, also during lighter activities such as undressing and dressing, see Figure 7.

The FPR of AF detection, determined in healthy subjects, has been used to assess SQIs developed for excluding segments with noise and artifacts [42]. A low-complexity AF detector was used [37], with an FPR of 1.4% determined on the Normal Sinus Rhythm Database. The poorest QRS detection performance was obtained during running, explaining the high FPR in about one third of the subjects, illustrated by Figure 5.

Inspired by the results presented in [34], suggesting that the electrode positions approximately equal to A and I are the least affected by motion artifacts, lead AI was chosen for signal quality assessment. Considering that the Piotrode was designed with the aim to reduce motion artifacts, lead AI is then likely the least favorable lead to use for such assessment. In other words, the gap in performance between the Piotrode and the Ambu is likely to widen when other electrode positions are subject to analysis.

Nonstandard single-lead placement has often been considered in several studies assessing signal quality, e.g., two electrodes placed on each side of the sternum rather close to the precordial leads $V_{1}$ and $V_{2}$ [25,27], one electrode placed at position A and the other electrode higher up than position I [23], two electrodes placed on the forearm at a distance of 8 cm [26], and different locations on the waist of two electrodes (textile sensors) [43]. In a few studies, the assessment has been based on standard lead systems, i.e., the standard 12-lead ECG [31] and the EASI lead system except lead AI [32]; it is unclear why this particular lead was disregarded.

Ideally, signal quality assessment should involve multiple leads suitably distributed on the body surface to provide a more complete characterization of signal quality. In the present study, simultaneous recording of more than one lead was not possible due to practical reasons. Consequently, only one lead for each type of electrode was recorded simultaneously. This limitation may be solved by repeating, either once or several times, the activity protocol so that one type of electrode is first used and then the other type [32]. However, the assessment would then be based on sequential recordings that may not allow a fair comparison of electrodes, one reason being that sequential recordings more likely exhibit larger differences with respect to the presence of motion artifacts than do simultaneous recordings. It should be noted that simultaneous recordings do not produce identical ECG signals since the two electrodes have to be placed edge to edge and thus pick up slightly different cardiac activity [23].

The proposed protocol was designed to account for everyday activities in which the subject remains dressed, except for undressing/dressing of the upper part of the body. The protocol resembles the one used in [32], however, the recording conditions differ since the subjects in that study were recorded during exercise stress testing (using printed, skin-mounted electrodes), and, accordingly, wore no garment on the upper part of the body throughout the protocol. Had the subjects worn a garment, the results would likely have been different due to electrostatic and mechanical noise induced by the garment. Studies assessing the signal quality of textile ECG electrodes usually involve subjects with a garment, e.g., a T-shirt, hosting the electrodes [25,31,43].

In the present study, several indices are employed for assessing signal quality of which QRS detection performance represents the most basic. This indirect SQI is easily determined since annotating QRS complexes is rather straightforward; the same observation applies to annotating AF episodes (which were absent in the present dataset). QRS duration has served as another indirect, though useful SQI, see, e.g., [12], however, its annotation is much more time-consuming as every single QRS complex of the dataset then need to be manually delineated. Out of the great number of SQIs proposed in the literature, the ensemble standard deviation ${\bar{\sigma }}_{e}$ is a classical measure of the noise level in ensembles with time-aligned events having similar morphology. The SQI $\bar{\rho }$, measuring the repeatability of waveform morphology contained in the PQRST interval, was chosen not only because it provides information which is complementary to ${\bar{\sigma }}_{e}$, but also because it has been found to offer a more accurate signal quality assessment than several other well-known SQIs [7]. Considering that motion artifacts represent a type of transient noise, yet another approach to assessing signal quality is to use a recently proposed deep learning based technique for detecting transient noise [44]; the percentage of signal segments without transient noise could then serve as SQI. Since a dataset much larger than the present one is needed for training of the deep network, this technique was not further considered.

A number of studies have argued that poor QRS detection performance is bound to influence an SQI [11,12,42]. However, the wavelet-based QRS detector, employed in the present study, has been evaluated on the MIT–BIH database with Se and PPV of 99.8%, thus implying that any significant reduction in performance is most likely related to poor signal quality, not detection performance. It should be noted though that QRS detection performance indices may be unfeasible when the annotation task becomes too time-consuming.

A number of limitations of the present study needs to be pointed out. The long-term properties of the Piotrode remains to be investigated as it has been shown that the artifact level may change during the course of a long-term recording [45]. Moreover, the signal quality of the Piotrode should also be studied in diagnostic terms to ensure that wave amplitudes, durations, and morphologies are accurate.

## 6. Conclusions

The signal quality of a novel wet ECG electrode, developed to reduce motion artifacts, is assessed. Compared to a commercially available, commonly used electrode, the Piotrode demonstrates better signal quality, and superior QRS detection. The Piotrode offers the potential to reduce the review burden, and accordingly the cost, associated with ambulatory monitoring.

## 7. Patent Application

The electrostatic and mechanical design of Piotrode electrode are subject to patent applications (SE2050287-8) and (SE2150147-3), respectively.

## Data Availability

The approval does not permit sharing.
